# MicroRNA Gatekeepers: Integrating Growth, Development, and Stress Adaptation in Cotton

**DOI:** 10.3390/plants15213268

**Published:** 2026-10-23

**Authors:** Jiarui Li, Xuan Wang, Jiawang Qin, Yifan Wu, Mengyao Wei, Chaofan Li, Yanfeng Jia

**Affiliations:** Xinjiang Key Laboratory of Biological Resources and Genetic Engineering, College of Life Science and Technology, Xinjiang University, Urumqi 830046, China

**Keywords:** cotton, MicroRNA, developmental control, biotic stress, abiotic stress

## Abstract

As the primary cash crop for natural fiber, cotton develops under sophisticated endogenous genetic programs, with biotic and abiotic stresses continuously driving its adaptive evolution. MicroRNAs (miRNAs) are post-transcriptional regulators that modulate gene expression via target mRNA cleavage, translational repression, and mediation of DNA methylation. miRNAs target transcription factors and hormone signaling pathways to regulate cotton plant architecture and fiber development. In addition, miRNAs mitigate biotic and abiotic stresses through ROS, lignin, and hormone pathways, as well as by forming competing endogenous RNA (ceRNA) modules with long noncoding RNAs (lncRNAs). This review establishes an miRNA-centered regulatory framework that coordinates cotton growth, development, and stress resistance. A series of technologies, including multi-omics combined with machine learning, gene overexpression, STTM, MIM, MIGS, CRISPR editing, foliar dsRNA spraying, and bionanocarriers, are proposed to facilitate the breeding of cotton varieties with high yield, superior fiber quality, and broad-spectrum stress tolerance.

## 1. Introduction

Cotton (*Gossypium* spp.) is the world’s predominant natural fiber crop and an important source of edible oil [1]. Featuring unique genomic features and fiber developmental characteristics, cotton represents an ideal model plant for dissecting genome evolution, polyploidization mechanisms, and the regulation of cell elongation [2]. Cotton growth and development are governed by precisely timed and highly ordered ontogenetic programs, which cover key biological processes including vegetative growth, reproductive maturation, the juvenile-to-adult phase transition, floral induction, and fiber elongation. A broad spectrum of biotic challenges, including fungal, viral, and insect attacks, as well as abiotic stresses such as salinity, drought, and heavy metal toxicity, act as major environmental forces driving adaptive evolution and shaping stress-resistance strategies in cotton [3]. To adapt to variable growing environments, cotton coordinates growth, development, and stress responses via multi-layered and sophisticated regulatory networks, involving multiple regulatory dimensions such as gene expression control, hormone signaling crosstalk, anthocyanin accumulation, and epigenetic modifications [4,5]. As core post-transcriptional regulators, microRNAs (miRNAs) orchestrate these complex regulatory networks and exert indispensable functions in cotton developmental regulation and stress resistance [6].

miRNAs are endogenous single-stranded noncoding small RNAs of 21-24 nucleotides (nt). They modulate the expression patterns of target genes at both transcriptional and post-transcriptional levels through target mRNA cleavage, translational repression, and DNA methylation [7,8,9,10]. Numerous studies have revealed the regulatory mechanisms underlying plant growth-development and stress resistance from the perspective of miRNAs [6,7,11,12]. Similar to other major crops, cotton miRNAs have been extensively investigated. However, the available reviews on cotton miRNAs only separately describe either cotton growth and development or stress responses, lacking an integrated analysis connecting these two processes [13,14]. Therefore, building upon existing reviews on plant miRNAs, this review systematically illustrates the core regulatory concept of miRNAs acting as molecular “gatekeepers”. Unlike earlier reviews that mostly focused on miRNA identification and the cataloging of isolated biological processes, this work integrates the latest research evidence from a cross-cutting perspective of development and stress responses. We comprehensively summarize how miRNAs govern cotton growth and development, as well as the response and adaptation mechanisms of cotton to biotic stresses including fungi, viruses, and insect pests, and abiotic stresses such as salinity, drought, high temperature (HT), heavy-metal toxicity, and nutrient imbalance. We further dissect the miRNA-mediated regulatory network underlying growth-defense trade-offs. This review also discusses the translational prospects of miRNA-oriented genetic improvement strategies for breeding stress-tolerant cotton varieties. It is anticipated to provide an integrative theoretical framework and biotechnological approaches to address the challenges facing cotton production under climate change and advance the sustainable development of the cotton industry.

## 2. Roles of miRNAs in Developmental Control of Cotton

**Figure 1:**
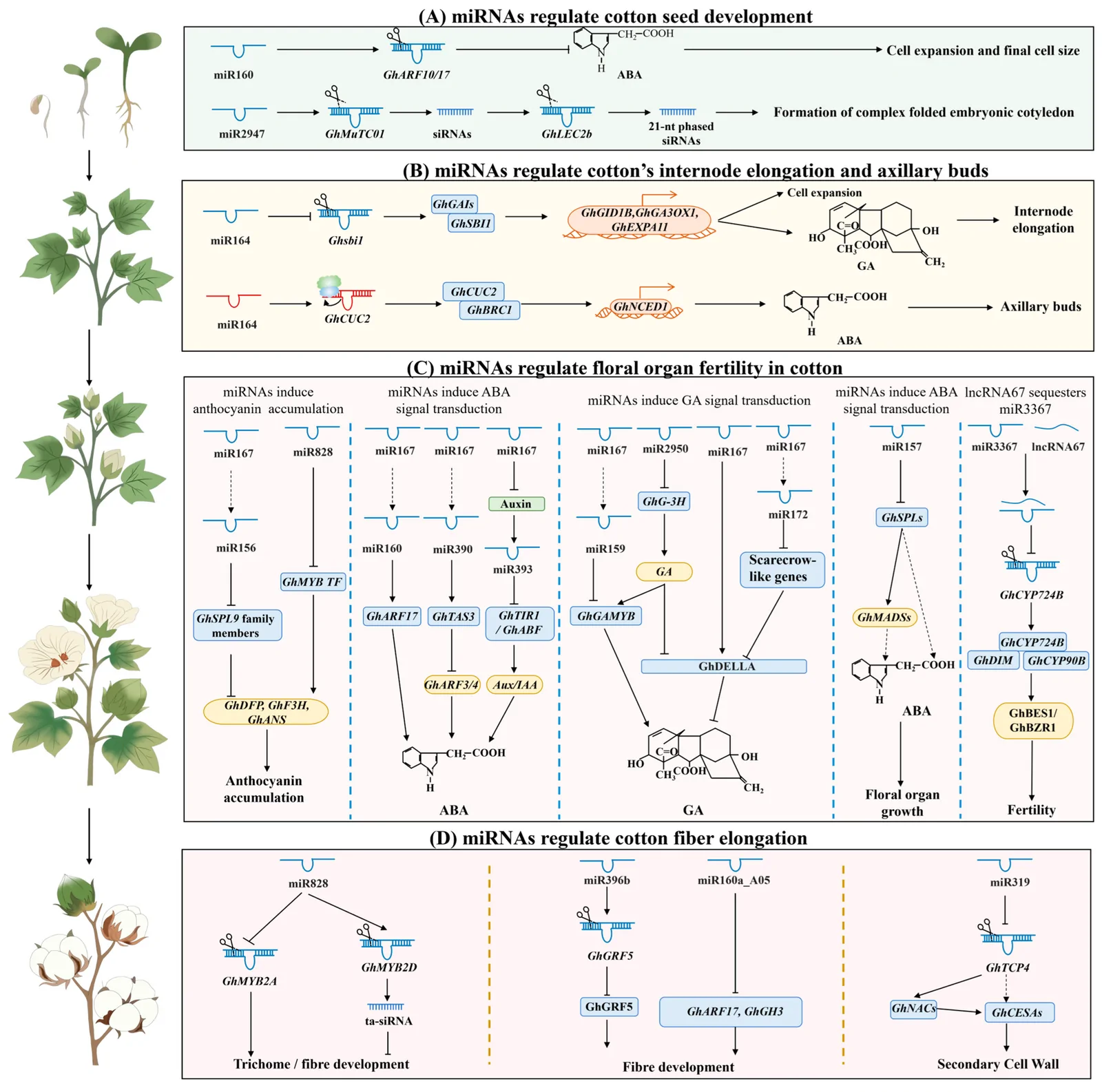
Overview of molecular mechanisms underlying miRNA-mediated regulation of cotton growth and development. miRNAs modulate cotton developmental events, including (**A**) seed development, (**B**) internode elongation and axillary bud outgrowth, (**C**) floral organ fertility and (**D**) fiber cell elongation, via phytohormone-mediated, anthocyanin-associated and TF-dependent regulatory modules. Solid arrows indicate positive regulation, blunt-ended lines indicate inhibition, and dashed arrows represent regulatory relationships that remain to be fully elucidated. Blue denotes mRNA cleavage and red indicates translational repression.

Cotton undergoes vegetative and reproductive growth, two fundamental developmental processes coordinately controlled by intrinsic genetic programs and environmental cues [15]. miRNAs precisely modulate cell proliferation and differentiation. By targeting transcription factors (TFs) and hormone signaling cascades, they serve as central hubs linking developmental signals with gene expression. Moreover, miRNA families exert critical functions in governing the programmed transition from vegetative to reproductive growth, mediating the orderly switching and dynamic balance between these two developmental stages [16] (Figure 1, Table 1).

### 2.1. miRNA Caretakers Governing Cotton Seed Development

Seeds constitute the primary harvestable organs of crops, with seed size and grain weight serving as key yield-determining traits. Meanwhile, cotton fiber cells initiate from ovule epidermal cells, so seed development exerts profound influences on fiber elongation, yield, and quality [17,28]. miR160 targets and regulates *ARF* to activate the ABA signaling pathway. It modulates seed coat growth by directly or indirectly regulating seed development-related genes, thereby determining seed size [17]. In parallel, the miR2947-*MuTC01* regulatory module participates in cotton embryogenesis and morphogenesis to finely control embryonic folding. Specifically, miR2947 targets and mediates the cleavage of *MuTC01* transcripts in *Gossypium raimondii*, which triggers the production of secondary small RNAs. The resulting 22 nt siRNAs target the key embryonic development gene *LEC2b* and induce mRNA cleavage, which in turn induces the biogenesis of 21 nt phased siRNAs (phasiRNAs), and these phasiRNAs ultimately regulate embryonic folding phenotypes [18].

### 2.2. miRNA Managers of Internode Elongation and Axillary Bud Development in Cotton

Diverse miRNAs precisely regulate cotton vegetative development, including internode elongation and axillary bud formation. In the network governing cotton plant architecture and branching, miR164 shapes plant architecture by targeting and repressing the NAC TF gene *GhSBI1*. A target-site mutation allows *GhSBI1* to evade miR164-mediated cleavage and overexpress. GhSBI1 interacts with DELLA proteins to inhibit gibberellin (GA) biosynthesis and signaling, shortening fruiting branch internodes and forming compact plant architecture suitable for dense planting [19]. Meanwhile, the miR164-*GhCUC2*-*GhBRC1*-*GhNCED1* module mediates axillary bud initiation, promotes abscisic acid (ABA) accumulation in axillary buds, and inhibits lateral branch elongation, also promoting compact cotton architecture [20]. These results lay important theoretical and molecular bases for improving cotton plant architecture and breeding high- and stable-yield cultivars.

### 2.3. miRNA Balancers Governing Floral-Organ Fertility in Cotton

Floral organ formation and fertility are core determinants of cotton yield and reproductive efficiency, and this process is precisely regulated by diverse miRNAs [23,29]. High-throughput sequencing analyses show that multiple differentially expressed miRNAs in cotton participate in vital reproductive developmental processes, such as hormone-mediated flowering time regulation and cytoplasmic male sterility, by targeting key functional genes [30,31]. The miR157-*SPL* regulatory module modulates MADS-box TFs and auxin signal transduction, and participates in floral organ development and ovule formation in cotton [21]. In contrast, the miR3367-lncRNA67-*GhCYP724B* pathway directly affects cotton male fertility by regulating brassinosteroid (BR) biosynthesis, thereby inducing male sterility [22]. Furthermore, multiple miRNAs associated with miR167 form an intricate regulatory network to coordinately govern cotton floral organ and fiber development. Specifically, miR156 and miR828 regulate anthocyanin biosynthesis by repressing *SPL9* family members and MYB TFs, and accumulated anthocyanin scavenges reactive oxygen species (ROS) and consequently influences floral organ and fiber development. miR156, miR390, miR160, miR167 and miR393 collectively participate in auxin signaling regulation. As the central hub of this network, miR167 negatively regulates auxin signaling by targeting *ARF6/8* and interacts with multiple miRNAs. miR159, miR2950, and miR172 are involved in GA signaling, acting on *GAMYB*, key GA biosynthetic enzymes, and Scarecrow-like family genes to mediate the GA signaling pathway. Aux and GA signaling pathways act synergistically with the anthocyanin metabolic pathway to regulate floral organ and fiber development [23].

### 2.4. miRNA Coordinators of Cotton Fiber Elongation

Cotton is the most important natural fiber crop worldwide, and fiber development is regulated by intricate molecular mechanisms, in which miRNAs can finely modulate fiber elongation at different developmental stages. miRNA sequencing was conducted on wild-type upland cotton and its fuzzless/lintless mutant, identifying 111 known miRNAs and 2 novel miRNAs involved in the regulation of fiber initiation [32]. Additionally, miRNA sequencing analysis of *Gossypium hirsutum* TM-1 and its fiberless mutant identified 27 conserved miRNAs and 4 novel miRNAs that are closely associated with fiber development [33]. Guan et al. confirmed that miR828 and miR858 affect fiber initiation by regulating *GhMYB2* in cotton [24]. Similarly, miR396b directly targets *GRF5* to positively regulate fiber growth [25]. In contrast, miR160a_A05 modulates fiber development by repressing the expression of *ARF17* and *GH3* [26]. The miR167-*ARF* regulatory module acts as a core hub to precisely control the proliferation and elongation of cotton fiber cells in a dose-dependent manner [34]. Cao et al. further illustrated that the expression level of *GhHOX3* is rapidly upregulated after fiber initiation, and GA promotes fiber cell elongation at least partially via *GhHOX3*. Meanwhile, the abundance of miR319 decreases, and the expression of its target gene *GhTCP4* and other TCP family genes increases gradually. TCP proteins inhibit the function of GhHOX3 and facilitate secondary cell wall (SCW) biosynthesis, thereby mediating the transition of fibers from the elongation stage to the secondary wall thickening stage [27].

Taken together, these developmental pathways demonstrate that miRNAs form a multilayered regulatory network governing cotton growth and development. Among them, miR156/157-*SPL*, miR164-*NAC* and miR167-*ARF* act as conserved core hubs spanning vegetative and reproductive development. These modules are activated spatiotemporally in specific tissues such as meristems, ovules, and fibers, and regulate phenotypes in a dose-dependent manner. Nevertheless, current research has notable limitations. Most studies focus on fiber and aboveground plant architecture traits, while the miRNA mechanisms underlying seed endosperm, root, and canopy development remain poorly characterized. Functional evidence for many miRNAs is obtained only from a single genotype, a single developmental stage, or laboratory culture conditions. Several studies have even reported opposite regulatory effects for the same miRNA; such discrepancies often arise from differences in genetic background, sampling time, and experimental environments. Furthermore, the synergistic and antagonistic interactions among miRNAs during the vegetative-to-reproductive transition have not been systematically deciphered. Future research, combining microarray and in situ hybridization techniques to dissect the mechanistic links between miRNA-target modules and hormone signaling pathways across multiple genetic backgrounds and time-series gradients, will provide theoretical support for molecular breeding of high-yield and high-quality cotton varieties.

## 3. miRNA-Mediated Orchestration of Biotic Stress Signaling in Cotton

**Figure 2:**
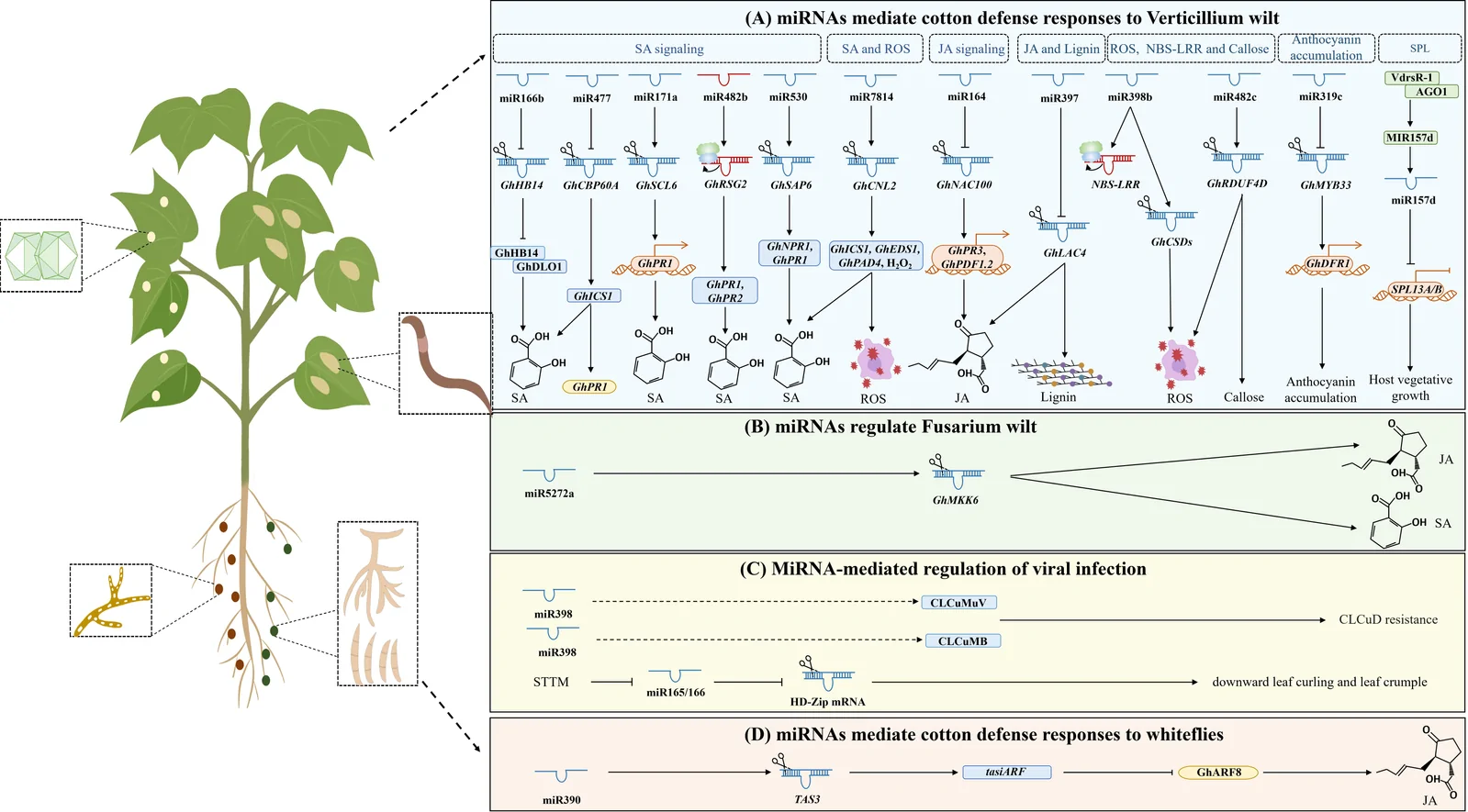
miRNA-mediated defense networks in cotton against biotic stresses. miRNAs modulate cotton defense against (**A**) Verticillium wilt, (**B**) Fusarium wilt, (**C**) virus infection, and (**D**) whitefly infestation via SA-, JA-, and ROS-dependent signaling cascades, as well as functional metabolites including lignin, callose, and anthocyanin. Solid arrows indicate positive regulation, blunt-ended lines indicate inhibition, and dashed arrows represent regulatory relationships that remain to be fully elucidated. Blue denotes mRNA cleavage and red indicates translational repression.

Cotton often suffers from biotic stresses such as fungi, viruses, and insect pests that impair yield and quality [35]. As post-transcriptional regulators, miRNAs govern cotton immunity, and dissecting such networks benefits resistance breeding (Figure 2, Table 2).

### 3.1. Cotton miRNA Reprogramming and Bidirectional sRNA Communication Under Fungal Stress

#### 3.1.1. miRNA-Mediated Interactive Responses of Cotton to *V. dahliae*

*Verticillium dahliae* (*V. dahliae*) is a hemibiotrophic soil-borne fungal pathogen that infects plants via roots, colonizes and proliferates within the vascular system, and ultimately induces wilting symptoms in host plants. Upon infection by *V. dahliae*, cotton responds to Verticillium wilt stress by modulating pathways involving salicylic acid (SA), jasmonic acid (JA), ROS, NBS-LRR proteins, lignin, callose, and anthocyanin, as well as SPL TF pathways. For instance, miR166b [36], miR171a [37], miR319b [38], miR477 [39], miR482b [40], and miR530 [41] cleave their downstream target genes *GhHB14*, *GhSCL6*, *GhTCP4-like*, *GhCBP60A*, *GhRSG2*, and *GhSAP6*, mediating cotton defense against Verticillium wilt via the SA signaling pathway. Other studies have revealed that miR7814 modulates the cleavage efficiency of *GhCNL2*, promotes the expression of *GhICS1*, *GhEDS1*, and *GhPAD4*, and thereby regulates the levels of SA and ROS [42]. Parallel to the SA pathway, miR164 represses *GhNAC100* expression by cleaving target mRNA, relieving its inhibitory effect on JA signaling and activating JA-mediated defense responses [43]. miR398b alleviates the cleavage of *GhCSD1*, *GhCSD2*, and *GhCCS1* to maintain ROS homeostasis in plants [44]. Infection by *V. dahliae* blocks miR482c-mediated degradation of *GhRDUF4D* mRNA, leading to significant upregulation of *GhRDUF4D*. As an E3 ubiquitin ligase, GhRDUF4D facilitates substrate ubiquitination, triggers ROS burst and callose deposition, and effectively inhibits pathogen colonization and spread [45]. VdsRNA derived from *V. dahliae* targets the miR157d precursor MIR157d. In cotton infected by *V. dahliae*, this targeting effect elevates the accumulation of miR157d. Differently upregulated miR157d targets and represses *SPL13A/B* to modulate cotton floral transition and consequently prolong the vegetative growth phase of plants [46]. Notably, this cross-kingdom sRNA communication operates between different species, which is distinct from the endogenous ceRNA regulatory module inside cotton cells.

#### 3.1.2. miRNA-Mediated Interactive Responses of Cotton to *Fov*

Fusarium wilt of cotton, triggered by *Fusarium oxysporum f. sp. vasinfectum* (*Fov*), is also a globally destructive soil-borne disease, in which the pathogen invades the roots and vascular tissues of susceptible cotton plants and ultimately causes severe wilting or even plant death [62]. Studies have shown that miRNAs identified from the mycelia and microsclerotia of *Fov* exhibit distinct expression patterns between these two developmental stages [63]. Notably, these miRNAs are endogenous to *Fov* rather than cotton miRNAs. Upon *Fov* infection in cotton, the GhMKK6-MAPK pathway is activated and triggers defense responses mainly through the SA and/or JA pathways. Meanwhile, miR5272a negatively regulates *GhMKK6* at the post-transcriptional level, forming a negative feedback loop to prevent excessive immunity and maintain homeostasis [49].

### 3.2. miRNA Crosstalk in Cotton-Virus Interactions

Cotton leaf curl disease (CLCuD) is a devastating viral disease caused by a complex of cotton leaf curl begomoviruses and transmitted by the whitefly Bemisia tabaci [64]. Studies have demonstrated that by adopting an endogenous miRNA-based regulatory strategy, overexpression of miR398 and miR2950 in upland cotton can target the genomes of Cotton leaf curl Multan virus (CLCuMuV) and its associated betasatellite (CLCuMB), and significantly enhance cotton resistance to leaf curl disease [50]. Similar to the pathogens causing CLCuD, cotton leaf crumple virus (CLCrV) is a double-stranded DNA virus capable of establishing systemic and persistent infection in cotton [65]. The cotton endogenous miR156b targets and represses the expression of multiple *SPL* family TF genes. Transient overexpression of miR156 in upland cotton via the CLCrV vector triggers plant developmental defects, including severe leaf wrinkling, downward curling of young leaves, and plant stunting [51]. Additionally, Zhang et al. constructed a CLCrV-mediated delivery vector for cotton CRISPR/Cas systems and verified that this viral vector enables efficient ABE base editing [66].

### 3.3. miRNA Cues Shaping Cotton-Pest Interactions

Cotton pests fall into three categories: piercing-sucking insects exemplified by whiteflies, plant-parasitic nematodes exemplified by root-knot nematodes, and chewing insects exemplified by cotton bollworms. miRNAs play pivotal roles in cotton anti-insect regulatory networks by precisely targeting key functional genes in plants. Among them, miR390 cleaves *TAS3* transcripts to produce tasiRNAs, which further modulate auxin response factor *ARF8* and form the linc1-miR390-TAS3 cascade module. This pathway significantly strengthens cotton resistance to whiteflies via crosstalk regulation that represses auxin signaling while activating JA signaling [52]. High-throughput sequencing has been adopted to profile miRNA expression during cotton-root-knot nematode interactions, identifying a large number of stress-responsive miRNAs [67,68,69]. However, existing studies on chewing pests such as cotton bollworms mostly focus on endogenous miRNAs of the pests rather than mechanistic analyses of host cotton miRNAs [70,71,72,73]. Overall, research progress varies across the three types of cotton pests, and cotton endogenous miRNAs serve as valuable molecular resources for deciphering pest-specific regulatory pathways and breeding broad-spectrum insect-resistant germplasm.

Cotton activates a complex miRNA reprogramming network upon biotic stress. Among these modules, miR164, miR390, and miR156/157-*SPL* act as conserved regulatory hubs functioning across developmental processes and multiple biotic stresses. They exhibit evident tissue specificity and temporal response characteristics in roots, vascular tissues, leaves, and other tissues. The defensive effects of a given miRNA are often genotype-dependent; opposite regulatory outcomes have even been reported across distinct cotton germplasms, pathogen strains, and experimental conditions. Such discrepancies usually arise from differences in genetic background, sampling time, inoculation dosage, and growth environments. Current studies still have notable limitations: most investigations are restricted to a single stress type, one cotton genotype, and a single time point. These studies tend to report individual miRNA-target modules independently and lack cross-comparison across different diseases and pests. Moreover, mechanistic research on cotton endogenous miRNAs against chewing insect pests remains scarce. Future research should systematically identify miRNAs and their target genes responsive to fungal, viral, and pest stresses, elucidate the molecular mechanisms by which miRNAs regulate cotton disease and insect resistance, explore shared miRNA-mediated cross-stress signaling pathways, and dissect the regulatory connections among distinct stress-response pathways. Site-specific modification of miRNAs or their target binding sites via gene editing technology enables precise remodeling of stress regulatory networks, which facilitates the creation of novel cotton materials with broad-spectrum disease and insect resistance without yield penalties. This will provide viable targets and theoretical support for molecular breeding of resistant cotton varieties.

## 4. miRNA-Mediated Abiotic Stress Responses in Cotton

**Figure 3:**
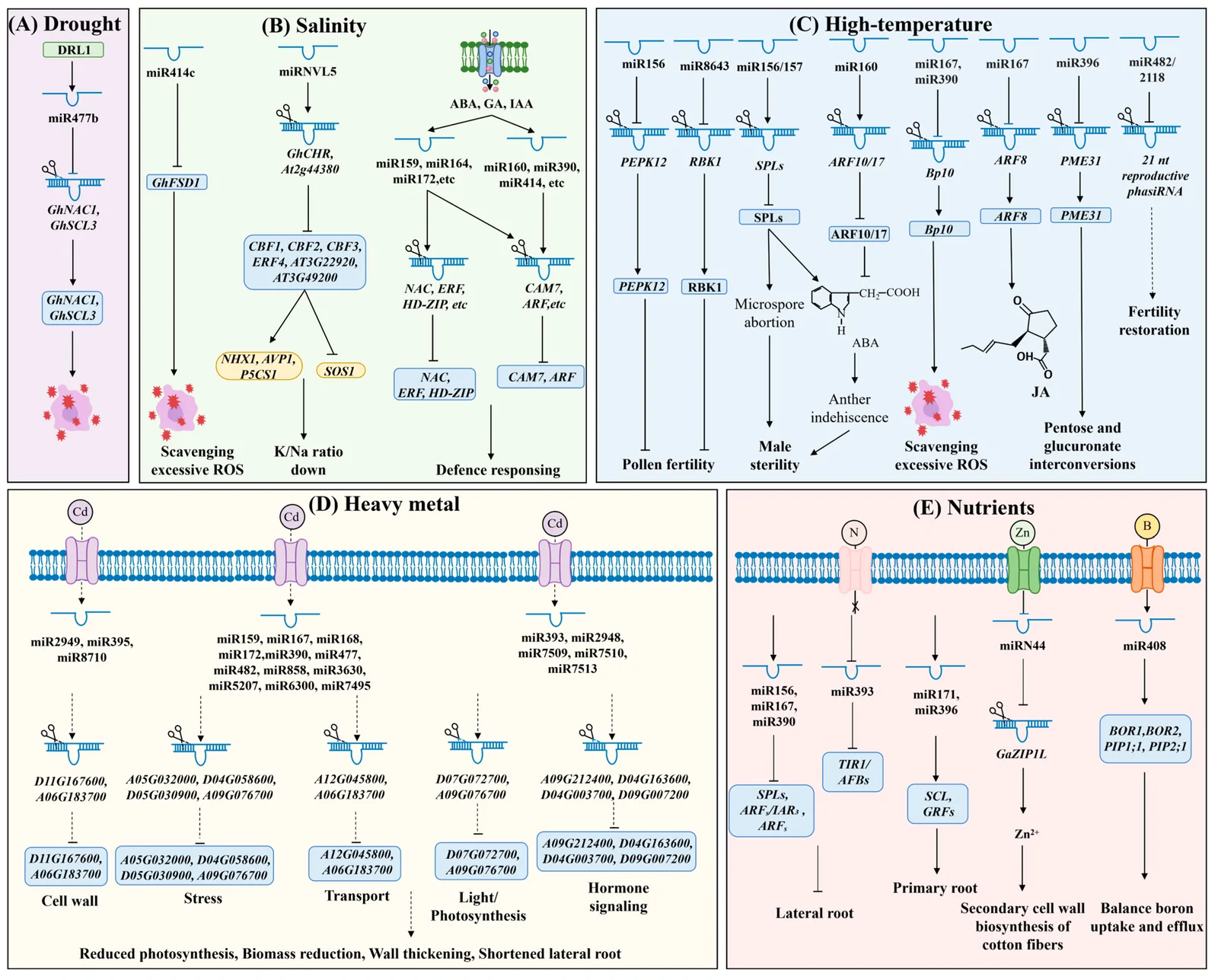
Overview of the regulatory network formed by miRNAs and their target genes involved in abiotic stress responses of cotton. Cotton miRNAs modulate diverse molecular pathways such as ROS signaling, K^+^/Na^+^ transport, ABA-JA hormone signaling, cell-wall remodeling, immune, and light-signaling pathways, as well as fiber secondary-cell-wall biosynthesis and boron uptake-efflux balance. These modules collectively control cotton responses to (**A**) drought, (**B**) salinity, (**C**) high temperature, (**D**) heavy metal stress, and (**E**) normal nutrient acquisition. Solid arrows indicate positive regulation, blunt-ended lines indicate inhibition, and dashed arrows represent regulatory relationships that remain to be fully elucidated. Blue denotes mRNA cleavage and red indicates translational repression.

Abiotic stresses such as drought, salinity, extreme temperatures, and heavy metals severely limit cotton growth and yield [74]. miRNAs act as core regulatory hubs bridging environmental signals and plant adaptive responses, as they modulate the expression of stress-responsive genes, the activity of antioxidant systems, ion homeostasis, and hormone signaling pathways to facilitate cotton acclimation to adverse environments (Figure 3, Table 2).

### 4.1. miRNA-Mediated Regulatory Modules for Drought Adaptation in Cotton

Drought is a major abiotic stress that drastically reduces cotton fiber yield and hinders the sustainable development of the cotton industry [75]. When cotton experiences drought stress, miRNAs modulate stress-responsive signaling pathways by targeting key functional genes, establish multi-layered regulatory networks, and cooperatively mediate plant adaptive responses to water deficit. A total of 379 drought-responsive miRNAs have been identified in drought-tolerant cotton germplasms [76]. High-throughput sequencing reveals numerous differentially expressed miRNAs between drought-tolerant and drought-sensitive cotton genotypes, which jointly confer drought resistance via sophisticated regulatory networks [77,78]. miR477b acts as a master regulator of cotton drought tolerance and directly targets two TFs, *GhNAC1* and *GhSCL3*. Drought-induced long non-coding RNA (lncRNA) DRL1 functions as a competing endogenous RNA (ceRNA) to competitively sequester miR477b and alleviate its repression on downstream target genes. Notably, this ceRNA regulatory module represents an endogenous regulatory pathway within cotton cells, which is distinct from cross-kingdom RNA communication occurring between different species. This ceRNA regulatory module activates multiple stress pathways, improves plant water use efficiency, stabilizes cell membrane structure, elevates antioxidant capacity, and ultimately greatly enhances drought resistance of cotton seedlings [53]. Apart from this regulatory mode, miRNAs also participate in drought defense by modulating epidermal wax biosynthesis. Recent studies have identified nine miRNAs targeting seven wax-synthetic *GhCER* genes, forming cascade regulatory pathways linked to ERF and MYB TFs [79]. These interconnected regulatory networks function synergistically, enabling cotton to coordinate growth and development with stress defense under drought conditions.

### 4.2. miRNAs: Fine-Tuning Cotton Responses to Salinity

Salt stress triggers a cascade of physiological responses including cellular dehydration, ion toxicity, and massive ROS accumulation, disrupting cellular homeostasis, reducing photosynthetic efficiency, disturbing metabolic pathways, and drastically impairing cotton yield and fiber quality. Numerous crop studies have verified that miRNAs act as core regulators governing plant salt stress responses [80,81,82].

High-throughput sequencing has shown that 12 miRNAs in cotton leaves are downregulated under high NaCl concentrations, whereas miRNA expression profiles in roots show substantial differences from those in leaves [83]. Additionally, salt stress induces miRNVL5 to cleave and repress *GhCHR*, thereby inhibiting stress-responsive TFs. This results in the downregulation of *SOS1* and the induction of several salt-responsive genes, ultimately triggering an imbalance in the K^+^/Na^+^ ratio and increasing the sensitivity of cotton to salt stress [55]. In contrast, miR414c is downregulated under salt stress, relieving repression of its target gene *GhFSD1*. Elevated expression of *GhFSD1* enhances the activity of SOD, accelerates ROS scavenging, and alleviates cell membrane damage, thereby improving salt tolerance in cotton [54]. Similarly, Zhan et al. found that the expression levels of novel-mir1, miR390, and miR393 gradually decrease under salt stress, which in turn activates signaling pathways involved in salt tolerance responses [84]. Chu et al. further confirmed that the miR390-*GhCEPR2* module regulates salt tolerance in cotton through the ABA signaling pathway [56]. In summary, cotton employs the complex ncRNA network involving miRNAs to coordinately regulate ROS scavenging, ion homeostasis, and ABA signal transduction under salt stress, providing key targets for molecular breeding of salt-tolerant cotton varieties.

### 4.3. miRNA-Mediated Homeostasis Regulation in Cotton Under Temperature Stress

Global warming brings two major extreme temperature stresses that hinder cotton output: low temperature (LT) and heat stress. LT includes chilling (0–15 °C) and freezing (<0 °C), which retard cotton vegetative growth, kill seedlings, and increase reproductive organ abscission. Flowering LT further causes heavy boll loss and poor fiber quality [85]. By contrast, flowering heat (day > 35 °C, night > 28 °C) easily induces anther male sterility and sharp yield reduction [86,87]. High-throughput sequencing analyses reveal that miRNAs exhibit obvious developmental-stage specificity in response to heat stress, and they target functional genes involved in multiple pathways including energy metabolism, hormone signal transduction, and cell cycle regulation [88]. Wang et al. identified 63 known miRNAs and 105 novel miRNAs in cotton, all of which are differentially expressed under both high- and low-temperature stress [89]. Previous studies have shown that the induction of miR160 under HT stress is genotype-dependent. MiR160 is up-regulated by HT in the heat-sensitive cotton line H05, whereas its expression is repressed in the heat-tolerant line 84021. Transgenic experiments have verified that miR160 targets and represses *ARF10/17*, which abnormally activates the auxin signaling pathway and ultimately triggers anther indehiscence. Meanwhile, HT suppresses the transcription of miR156/157 in both cotton lines. This leads to elevated expression of downstream *SPL* family genes and further induces microspore abortion. These two pathways function synergistically to induce male sterility in cotton anthers [57]. Analogously, heat stress downregulates miR156 and miR8643, relieving their cleavage repression on the membrane receptor-like kinase genes *PERK12* and *RBK1*. The markedly increased transcript abundance of these two genes disrupts pollen fertility homeostasis. In contrast, heat-induced miR167 and miR390 cleave their target gene *Bp10*, repressing genes related to the ROS scavenging pathway and disturbing intracellular oxidative homeostasis. Among them, miR167 can further target and cleave the key auxin signaling gene *ARF8*, altering the transcript abundance of downstream targets and disrupting JA metabolic balance. This eventually causes abnormal anther dehiscence and pollen abortion through uncharacterized molecular mechanisms. Additionally, heat-responsive miRNAs and downstream triggered sRNA cascades participate in the regulation of cotton fertility restoration. Members of the miR482/2118 superfamily trigger the biogenesis of 21-nt reproductive phasiRNAs, which further modulate the cotton fertility-restoration process. HT also activates the miR396-*PME31* module to remodel the metabolism of pentose and glucuronate interconversions [58]. To date, the miRNA regulatory network under heat stress in cotton has been systematically characterized, whereas comprehensive research on the molecular regulatory mechanisms mediated by miRNAs under cold stress is still lacking. Collectively, miRNAs modulate cotton adaptability to extreme HT and LT by governing core physiological processes including auxin signaling, ion homeostasis, and ROS scavenging, which provides vital molecular targets for breeding temperature-tolerant cotton germplasm.

### 4.4. miRNA Crosstalk in Cotton Under Heavy Metal Stress

Lead and cadmium are two highly toxic heavy metals that readily accumulate in soil and are absorbed by cotton, disrupting normal growth and development by damaging photosynthetic pigments and reducing chlorophyll content [90]. Studies have shown that miRNAs and their target genes exhibit tissue-specific and concentration-dependent expression patterns under lead stress [91]. Zeng et al. identified 35 miRNAs responsive to cadmium (Cd) stress in Cd-tolerant and Cd-sensitive cotton materials, including miR159, miR167, miR172, and miR390, which target genes associated with cell wall, stress response, ion transport, photosynthesis, and hormone signaling pathways, thereby modulating the phenotypic responses of cotton to Cd stress [92]. However, current studies on miRNA-mediated responses to heavy metal stress are mainly based on high-throughput sequencing, with relatively insufficient mechanistic dissection and functional verification. Further in-depth dissection of such regulatory networks will provide a critical theoretical foundation and molecular resources for molecular design breeding and targeted genetic improvement of cotton, as well as the breeding of specialized cotton varieties for ecological restoration with strong tolerance to both lead and cadmium and high heavy metal accumulation capacity.

### 4.5. miRNA Regulatory Circuits Modulating Nutrient Imbalance in Cotton

Cotton growth, yield formation and fiber quality development are highly dependent on adequate supplies of macronutrients including nitrogen, phosphorus, potassium, calcium, magnesium and sulfur, as well as micronutrients such as boron, iron, copper, zinc, manganese and nickel [93,94]. Under variable supply levels of nitrogen [59,95], phosphorus and potassium [96], miRNAs precisely regulate nutrient use efficiency and environmental adaptability of cotton by positively or negatively modulating target gene expression and cooperating with the regulatory networks of TFs and transporters. Under nitrogen-deficient stress, upregulated miR156, miR167, and miR390 separately target and repress *SPLs*, *ARFs/IAR*_3_, and *ARFs* to trigger lateral root initiation. Nevertheless, downregulated miR393 relieves its cleavage-mediated repression of auxin receptors *TIR1/AFBs*, and this inhibitory effect dominates, ultimately suppressing lateral root development. Meanwhile, highly accumulated miR171 and miR396 transcripts negatively regulate *SCL* and *GRFs*, respectively, and synergistically promote the longitudinal elongation of primary roots, enabling cotton to form an adaptive root architecture characterized by fewer lateral roots and elongated primary roots under low-nitrogen conditions [59]. The micronutrient zinc also acts as an essential factor sustaining normal cotton growth and development. Existing research has confirmed that miR44 represses the transcription of the novel zinc transporter gene *GaZIP1L*, modulating zinc transport efficiency during ovule development to supply sufficient zinc for normal embryonic growth [60]. Beyond balancing the supply and demand of nutrients in plants, miRNAs can also alleviate phytotoxicity induced by excessive accumulation of micronutrients. Taking boron toxicity as an example, high-boron treatment markedly induces the upregulation of miR408 in cotton. By modulating copper ion homeostasis and cell wall lignification, this miRNA indirectly alters the transcript levels of boron transporters *BOR1/BOR2* and aquaporins *PIP1;1/PIP2;1*, establishing a miR408-copper homeostasis-boron transport cascade regulatory pathway to improve cotton tolerance to boron toxicity [61]. In summary, miRNAs precisely mediate the transmembrane transport and internal homeostasis of all essential macronutrients and micronutrients, serving as molecular switches and central regulatory hubs within the regulatory network governing efficient nutrient utilization in cotton.

Drought, salinity, extreme temperatures, and heavy-metal toxicity constrain cotton growth, yield, and fiber quality. As core environmental-response hubs, miRNAs regulate functional genes involved in wax synthesis, the SOS pathway, ROS scavenging and hormone signaling, and also mediate heavy metal detoxification. Summarizing studies on diverse abiotic stresses, modules including miR390, miR156/157 and miR167 serve as conserved regulatory hubs responsive to multiple abiotic stresses. These miRNAs exhibit pronounced tissue specificity and temporal expression patterns, and their regulatory effects on stress responses are genotype-dependent. Opposite regulatory outcomes have even been observed under different experimental conditions such as distinct cotton germplasms, stress intensities, and treatment durations. For instance, heat stress induces the upregulation of miR160 in heat-sensitive cotton line H05 while repressing its expression in heat-tolerant line 84021, which leads to divergent activation status of auxin signaling and contrasting anther phenotypes between the two genotypes. Such discrepancies largely arise from differences in genetic background, sampling time, stress dosage, and growth environments. Current research still has notable limitations: most findings are merely correlative outputs from high-throughput sequencing and lack rigorous in vivo genetic functional validation. The miRNA regulatory pathways associated with cold stress, nutrient deficiency, and lead toxicity in cotton remain poorly characterized. Most studies independently report individual miRNA-target modules, lacking cross-comparison across different categories of abiotic stresses. Future research can employ overexpression, knockout, and gene-editing tools to dissect key miRNAs and their ceRNA/phasiRNA cascades. Combined multi-omics analysis and field trials will uncover conserved multi-stress-responsive miRNA modules and distinguish shared and stress-specific regulatory mechanisms. Molecular breeding using validated stress-tolerant and nutrient-use-efficient miRNAs together with their target genes can develop cotton varieties with broad-spectrum stress resistance and high nutrient use efficiency, advancing cotton stress breeding from simple correlation analysis toward mechanistic interpretation and field application.

## 5. Conserved miRNA Regulators in Cotton: Integrating Responses to Growth-Defense

**Figure 4:**
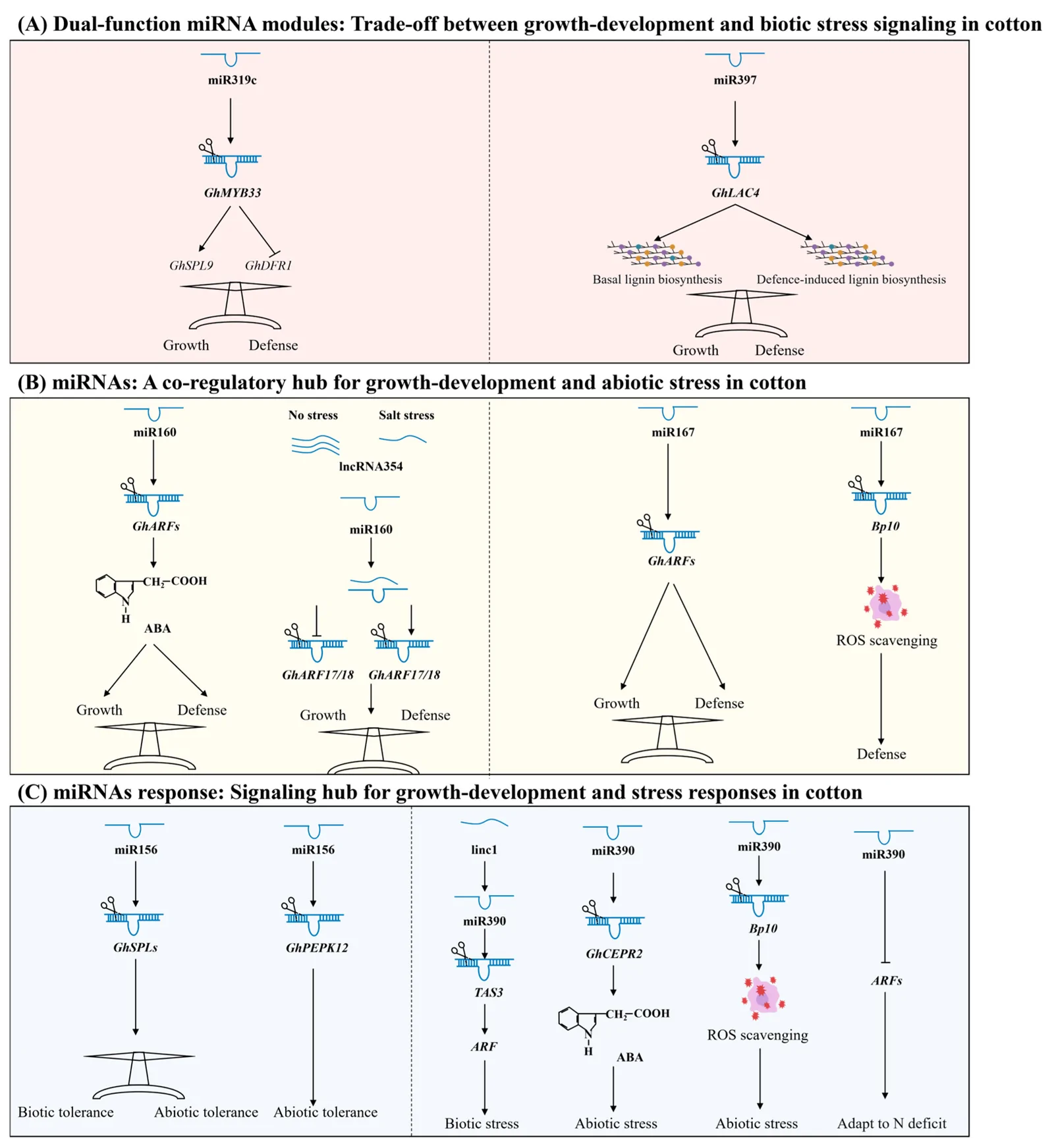
Schematic model illustrating regulatory networks of growth and stress responses associated with miRNA-hijacking effects in cotton under biotic and abiotic stresses. Solid arrows indicate positive regulation, blunt-ended lines indicate inhibition, and dashed arrows represent regulatory relationships that remain to be fully elucidated. Blue denotes mRNA cleavage and red indicates translational repression.

As pleiotropic regulators, miRNAs coordinate crop growth and immune defense and participate in both plant development and biotic stress responses (Figure 4).

### 5.1. Dual-Function miRNA Modules: Trade-Off Between Growth-Development and Biotic Stress Signaling in Cotton

Plants frequently exhibit growth inhibition upon pathogen infection, and this phenomenon is known as the growth-defense trade-off. miRNAs represent one of the core factors modulating crop growth and immune defense by regulating the expression of target genes such as TFs and mediating crosstalk with phytohormone pathways [12]. Multiple miRNA modules in cotton exert dual functions, and the same miRNA cascade participates in normal plant growth and development as well as immune responses against *V. dahliae*, forming such dual-function regulatory modules. miR319c targets *GhMYB33* and modulates downstream growth- and defense-related genes, including *GhSPL9* and *GhDFR1*, to balance cotton growth and disease resistance [47]. The dual-functional miR397-*GhLAC4* module governs dynamic lignin metabolism. It maintains basal lignin synthesis to support normal plant growth under non-stressed conditions. Upon *V. dahliae* infection, it induces massive lignin production via *GhLAC4* to strengthen cotton defense against the pathogen [48].

### 5.2. miRNAs: A Co-Regulatory Hub for Growth-Development and Abiotic Stress in Cotton

Abiotic stress can induce a fitness cost between plant growth and stress resistance. As core regulatory hubs, miR160 and miR167 rely on ARF-targeted networks to exert pleiotropic regulation in a dose-dependent and spatiotemporally specific manner, participating in the coordinated regulation of growth and stress responses. Targeting ARF family genes, miR160 participates in ABA and auxin signaling, fulfilling multiple functions in seed coat development, fiber formation, HT responses, and salt stress adaptation [17,26,57]. Under salt stress, lncRNA354 can sequester miR160b to de-repress *ARF17/18*, coordinating root development and salt tolerance [97]. Similarly, the miR167-*ARF* module is also pleiotropic. It is involved in auxin and gibberellin signaling and anthocyanin metabolic networks, regulating the proliferation and elongation of cotton fiber cells in a dose-dependent manner [23,34]. Meanwhile, it responds to stresses such as HT and nitrogen deficiency, and balances reproductive fertility and root plasticity via auxin-JA crosstalk and ROS homeostasis [58,59]. These two types of miRNAs can switch regulatory outputs across different developmental stages and stress environments to dynamically coordinate growth maintenance and stress resistance.

### 5.3. miRNAs Response: Signaling Hub for Growth-Development and Stress Responses in Cotton

Beyond participating in the regulatory processes associated with the fitness cost of growth and stress responses, miR156 and miR390 also exhibit pleiotropic features and respond to diverse biotic and abiotic stresses. Centered on SPL pathways, miR156 coordinates growth and stress resistance in a spatiotemporally specific manner. Viral infection, HT, and nitrogen limitation alter the transcript abundance of miR156, modulating SPL and downstream *PERK12* to participate in microspore development, pollen fertility maintenance, and lateral root formation [51,57,58,59]. miR390 mediates responses to whitefly infestation, salt stress, HT, cadmium stress, and nitrogen deficiency via the linc1-miR390-TAS3 cascade, ABA signaling, and redox homeostasis pathways. This module switches auxin and JA signaling outputs under different environments to differentially regulate root development and stress resistance, demonstrating the spatiotemporal characteristics of miRNA regulatory networks [52,56,58,59,92].

Collectively, these miRNA-centered regulatory modules finely coordinate Aux-JA-ABA crosstalk, lignin and anthocyanin metabolism, ROS homeostasis, and root architectural plasticity, enabling cotton to dynamically adjust growth and defense strategies under variable environmental conditions. miR156, miR160, miR167, miR390, and other miRNAs serve as conserved regulatory hubs mediating the growth-defense trade-off in cotton. Their regulatory effects exhibit prominent tissue specificity and temporal response characteristics and are highly genotype-dependent. Opposite regulatory outcomes can even be observed under different experimental conditions, including distinct germplasms, stress intensities, and treatment durations. Such discrepancies are generally caused by confounding factors such as genetic background, sampling time, stress dosage, and growth environments. LncRNA-mediated ceRNA networks further increase the complexity of miRNA-dependent growth and stress responses, supporting flexible spatiotemporal regulation of cotton development and stress resistance. Current studies have certain limitations: most reports characterize individual miRNA-target modules separately, lacking cross-comparison between biotic and abiotic stresses; numerous high-throughput sequencing data only provide correlative evidence without systematic in vivo genetic functional validation. Future research can employ transgenic overexpression, gene knockout, and CRISPR-Cas9 editing to functionally validate core miRNA-target and miRNA-ceRNA regulatory cascades. Multi-omics integration will help dissect conserved and specific molecular modules underlying fiber development, *V. dahliae* resistance, and abiotic stress adaptation, prioritizing the molecular switch mechanism of miRNA-hijacking effect governing growth-defense trade-offs. Validated miRNA regulatory modules can be applied in molecular breeding to develop elite cotton germplasms with superior fiber quality, enhanced disease resistance and high nutrient-use efficiency, translating fundamental research on miRNA functions into cotton genetic improvement.

## 6. Future Outlook and Strategies

**Figure 5:**
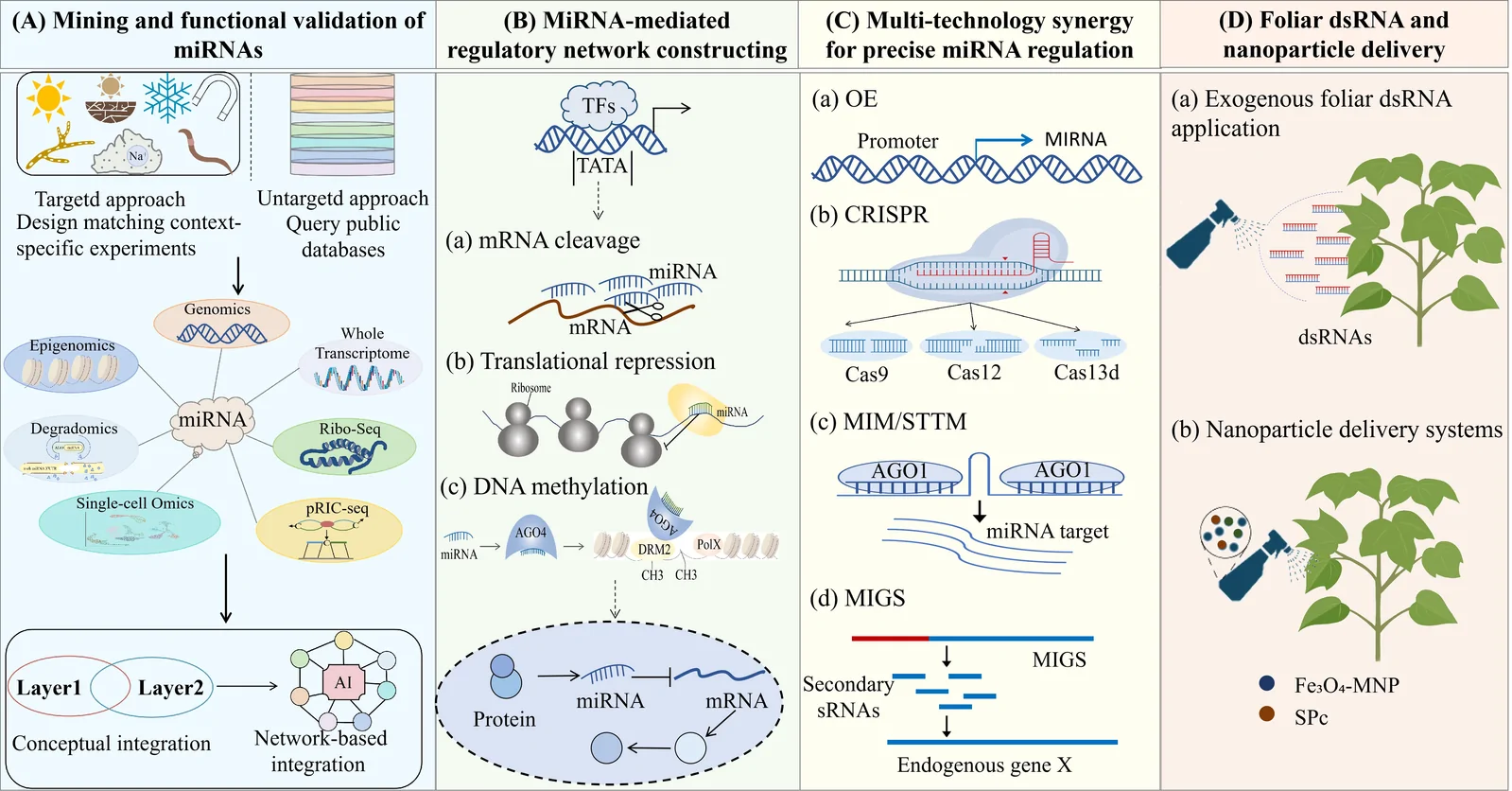
Comprehensive miRNA-based breeding strategies for developing disease-resistant cotton. (**A**) Mining and functional validation of miRNAs. (**B**) miRNA-mediated regulatory network construction. (**C**) Multi-technology synergy for precise miRNA regulation. (**D**) Foliar dsRNA and nanoparticle delivery. MIM: mimicry. STTM: Short Tandem Target Mimicry. MIGS: miRNA-induced gene silencing. dsRNA: Double-stranded RNA. pRIC-seq: Plant RNA in situ conformation sequencing. Ribo-seq: Ribosome profiling.

Biotic and abiotic stresses impair cotton growth, yield, and fiber quality and limit industrial sustainable development. A multidimensional technical system based on miRNA regulatory networks is critical for improving cotton stress adaptability and agronomic performance. Combined multi-sequencing and machine learning have demonstrated the capacity to overcome the limitations of single-omics approaches in other species, enabling the identification of miRNA regulatory patterns, stress-responsive genes, and TF-miRNA-target pathways. Nevertheless, large-scale application of this computational strategy for dissecting cotton miRNA networks still awaits further experimental validation. Cotton miRNAs modulate fiber development and immunity through mRNA cleavage, translational repression, and RdDM-dependent epigenetic modification. Tools including miRNA overexpression, mimicry (MIM), short tandem target mimicry (STTM), MiRNA-Induced Gene Silencing (MIGS), and CRISPR editing facilitate precise miRNA functional regulation. Double-stranded RNA spraying coupled with bionanotechnology has shown disease-control potential in various crops. However, it remains at the laboratory research stage in cotton and faces challenges before mature field application. Collectively, integrated multi-omics analysis, mechanistic research, gene editing, and nanodelivery systems refine cotton miRNA regulatory theories, accelerate stress-tolerant cultivar breeding, and promote sustainable cotton industry development (Figure 5).

### 6.1. Omics-Driven Intelligent Algorithms for Systematic Mining of miRNAs and Target Genes

Advanced multi-omics approaches, including full-length transcriptome, translatome, polysome profiling, small RNA sequencing, Plant RNA in situ conformation sequencing (pRIC-seq) and degradome sequencing, facilitate studies on miRNA-governed cotton development and stress responses. Differential transposable-element amplification alters transcription and alternative splicing of cotton homologous genes, disrupts miRNA-target recognition, and drives interspecific divergence [98]. pRIC-seq generates genome-wide RNA interaction maps for diploid and tetraploid cotton and uncovers spatial RNA regulation [99]. Combined transcriptome-degradome analyses have identified 43 miRNA targets linked to cadmium tolerance [92]. Nevertheless, current cotton miRNA multi-omics datasets remain fragmented. Single-omics approaches produce false-positive results and fail to resolve spatiotemporal or cell-type-specific miRNA regulation, hindering mechanistic studies.

In *Arabidopsis*, integrated time-series small RNA and single-cell transcriptome data have built high-resolution TF-miRNA-gene networks and validated miRNAs as core regulators of developmental and stress responses [100]. Constrained by cotton’s complex allotetraploid genome and species-specific miRNA regulatory circuits, the single-cell analytical frameworks of TF-miRNA-gene networks established in *Arabidopsis thaliana* have not yet been fully constructed in cotton. Machine learning provides computational approaches for integrative multi-omics mining to screen candidate genes and favorable alleles governing cotton growth and stress resistance. In theory, this analytical strategy can take gene pleiotropy and developmental trade-offs into account to mitigate analytical bias, yet its practical performance in deciphering cotton miRNA regulatory networks still requires further experimental validation with more datasets.

### 6.2. Mechanism of miRNA-Mediated Target Gene Regulation in Cotton

Dissecting the regulatory interactions between cotton miRNAs and their target genes helps elucidate the molecular mechanisms underlying cotton growth and development, and provides a theoretical basis for agronomic-trait improvement and molecular breeding. mRNA cleavage represents the predominant post-transcriptional mode of miRNA action, which can be verified by degradome sequencing and 5′-RACE. Besides target-mRNA cleavage, miRNAs exert translational repression; they modulate lignin, flavonoid, and ROS pathways to regulate fiber development and biotic resistance responses. Meanwhile, miRNAs can activate RdDM-dependent epigenetic silencing to stabilize agronomic traits and enhance stress adaptability in cotton [101]. Nevertheless, the dynamic crosstalk among miRNA regulatory pathways remains unclear in cotton. PHAS-derived siRNAs have only been partially characterized at the omics level, and their full landscape awaits further exploration. RdDM-dependent heritability of stress memory has been reported in model plants, whereas sufficient in vivo functional experimental evidence is still lacking in cotton. Future studies may combine multi-time-point omics analysis with genetic manipulation to identify miRNA-target modules with potential for cotton breeding applications.

### 6.3. Multi-Technology Synergy for Precise Gene Functional Regulation: Overexpression, MIM, STTM, CRISPR, and MIGS

Major plant-miRNA functional tools include overexpression, MIM, STTM, MIGS, and CRISPR editing [102,103,104,105]. Precursor overexpression produces gain-of-function lines. In cotton, miR319c balances plant growth and Verticillium wilt resistance, while miR398b and miR482b regulate cotton immunity against *V. dahliae* [40,44,47]. Conversely, MIM and STTM inhibit mature-miRNA activity to de-repress target genes, while MIGS enables simultaneous multi-gene silencing [102,106]. STTM164 sequesters miR164, upregulates *GhCUC2*, and modulates lateral branch development and ABA accumulation in axillary buds [20]. Mimicking the endogenous biogenesis pathway of secondary siRNAs, MIGS enables simultaneous silencing of multiple genes, which is suitable for cotton antiviral research and the dissection of multi-gene regulatory networks [105]. Meanwhile, diverse CRISPR tools allow precise manipulation of miRNAs in plants, though the application of some systems remains to be validated in cotton. The MND-Cas9 system removes miRNA stem-loop structures to achieve complete gene knockout [66]. CRISPR/Cas12a mediates multiplex editing of miRNA precursors and miRNA binding sites [107,108]. RNA-targeted Cas13d mediates reversible and transient regulation at the post-transcriptional level [109]. Single-cell CRISPR combined with spatial transcriptomics facilitates cell-layer-specific functional analysis, advancing research toward causal validation at single-cell resolution [65].

Although these approaches enable gain- and loss-of-function screening as well as genome editing, most cotton studies adopt only a single experimental method without cross-validation. Multi-tool combined research at single-cell resolution is rarely reported. There is therefore an urgent need to establish an integrated analytical framework to decipher miRNA-centered regulatory networks governing cotton growth and stress resistance.

### 6.4. Exogenous Foliar dsRNA Application and Nanoparticle Delivery Systems

Combining exogenous foliar dsRNA spraying with bionanotechnology represents a promising strategy to improve cotton stress tolerance and advance sustainable green agriculture. Exogenously applied synthetic dsRNA is processed by plant Dicer-like enzymes to generate dsRNA-derived siRNAs. These siRNAs target pathogen transcripts, silence pathogenic genes, and attenuate fungal virulence. This RNA-based defense has been validated in *Arabidopsis thaliana* and tomato [110,111]. Nevertheless, relevant cotton research lags behind and encounters several practical obstacles. Naked dsRNA is environmentally labile and easily degraded, and cotton cells take up dsRNA inefficiently, greatly restricting the field performance of RNA-mediated disease control [112]. Bionanotechnology offers feasible, eco-friendly solutions to overcome these bottlenecks. It improves delivery of functional molecules, mitigates fungal-stress damage, and suppresses pathogen proliferation [113]. Zhang et al. designed an artificial miRNA (si-tsRVal) and loaded it onto Fe_3_O_4_ magnetic nanoparticles (MNPs) to form stable spherical complexes (MNP-si-tsRVal). Through foliar spraying, this complex efficiently delivers si-tsRVal plasmids into apple leaf cells. It suppresses the accumulation of tsRVal, which targets the apple disease-resistance gene *Malus domestica TRANSPORT INHIBITOR RESPONSE 1* (*MdTIR-1*), thereby enhancing apple resistance to early defoliation disease [114]. Yin et al. constructed a star polycation (SPc)-based nanocarrier delivery system. SPc-loaded miR393 can activate jasmonic acid and salicylic acid signaling pathways, repress the expression of auxin receptor genes, and induce massive accumulation of *PR1*, thereby enhancing tomato resistance to gray mold [115].

Collectively, this strategy combines dsRNA-triggered exogenous RNA silencing, endogenous miRNA-based regulation, and bionanotechnology, offering new insights for cotton molecular breeding and eco-friendly disease management. At present, the efficacy of dsRNA spraying and nanocarrier-mediated RNA delivery has mostly been verified only in laboratory experimental systems of other crops. These techniques are still under exploratory research in cotton and have not evolved into mature field-applicable technologies. Further optimization of dsRNA-spraying and nanodelivery techniques, as well as in-depth functional characterization of cotton miRNAs, is still required. The miRNA-based nano-RNA strategy may serve as a potential molecular tool for future green cotton production and provide technical alternatives to achieve efficient and sustainable improvement of stress tolerance and disease prevention.

## 7. Conclusions

miRNAs act as core molecular “gatekeepers” within regulatory networks and serve as key hubs linking developmental and stress signals, exhibiting pleiotropic functions: (I) governing the developmental processes of seeds, plant architecture, and fibers. (II) mediating cotton responses to biotic and abiotic stresses. (III) regulating growth-defense trade-offs via the miRNA-hijacking effect. Beyond highly conserved miRNA modules shared among land plants, cotton has evolved lineage-specific miRNAs (miR2947, miR44), and several conserved miRNA pathways have undergone neofunctionalization. Conserved modules including miR319-*TCP* and miR828-*MYB* have acquired cotton-specific roles in determining fiber cell fate. Moreover, distinctive cross-kingdom small-RNA regulatory events occur during cotton-*V. dahliae* interactions. These lineage-specific sRNA events underpin cotton-exclusive biological traits such as fiber development and pathogen adaptation, and their regulatory mechanisms cannot be directly inferred from research on model plants like *Arabidopsis*. Additionally, miRNA-based cotton genetic breeding faces multiple bottlenecks. Most regulatory modules lack in *planta* genetic functional evidence; the mechanisms of ceRNA-phasiRNA regulatory circuits under combined-stress conditions remain unclear; and gene-editing techniques also have certain application limitations. To address these challenges, this review constructs an miRNA-centered regulatory model and proposes corresponding research strategies: performing causal genetic validation of key regulatory cascades, mining functional genes by integrating multi-omics and machine learning, and conducting systematic field phenotypic evaluation. Future efforts should decipher the molecular switches underlying the miRNA-hijacking effect to provide theoretical support for breeding novel cotton materials that balance yield, fiber quality, and broad-spectrum stress resistance.

## Figures and Tables

**Table 1:** miRNAs involved in cotton growth and development.

Biological Outcome	miRNA	Developmental Stage	Target (s)	Assays for miRNA-Target Validation	Tissue	Direction of miRNA Expression	Species	Validation Method	Resistance Characteristics	Reference
Seed-coat/ovule cell expansion; seed size and fiber-yield regulation	miR160	Ovule developmental stage	*GhARF10*	5′-RACE/psRNATarget prediction	Ovule/seed coat	↑	*Gossypium hirsutum*	MIM	Positive	[17]
Cotyledon morphogenesis of cotton embryo; Seed development	miR2947	20 DPA ovule/embryo developmental stage	*MuTC01*	psRNATarget prediction/ 5′-RACE/ Degradome sequencing	Ovule-embryo tissue	↑	*Gossypium raimondii/Gossypium hirsutum*	CRISPR-Cas9	Positive	[18]
Fruiting-branch internode elongation and plant architecture; organ-boundary development	miR164	Floral bud/anther development stage	*GhSBI1*	psRNATarget prediction/ 5′-RACE/degradome sequencing	Shoot apex/axillary bud/fruiting-branch internode	/	*Gossypium hirsutum*	CRISPR-Cas9/OE	Negative	[19]
Axillary-bud outgrowth and lateral-branch development/branch length	miR164	Vegetative-reproductive transition/axillary-bud development stage	*GhCUC2*	psRNATarget prediction/ 5′-RACE/degradome sequencing	Axillary bud/shoot apex	/	*Gossypium hirsutum/Arabidopsis thaliana*	VIGS/OE	Negative	[20]
Floral-organ size, ovule and seed production/cell proliferation and cell expansion	miR157	Floral bud development stage	*GhSPL*	psRNATarget prediction/ 5′-RACE	Floral bud/petal, anther/ovule/root/leaf/hypocotyl	↑	*Gossypium hirsutum/Arabidopsis thaliana*	OE	Negative	[21]
Brassinosteroid biosynthesis, microspore abortion and cytoplasmic male-sterility/Anther-pollen development	miR3367	Flower bud development stage	*GhCYP724B*	psRNATarget prediction/ 5′-RACE	Flower bud	↑	*Gossypium hirsutum*	VIGS/CRISPR-Cas9	Negative	[22]
Floral organ, anther-pollen and fiber-ovule development/flower-boll	miR156	Flower bud development stage	*GhSPL9*	psRNATarget prediction	Floral organs/ovule/fiber/leaf	↓	*Gossypium hirsutum*	MIM	Positive	[23]
Fiber cell-fate determination and trichome-like cell development	miR828	Ovule and fiber developmental stages	*GhMYB2*	psRNATarget prediction/ 5′-RACE	Ovule/fiber/leaf	↑	*Gossypium hirsutum/Arabidopsis thaliana*	asiRNA	Positive	[24]
Fiber cell-fate determination and trichome-like cell development	miR858	Ovule and fiber developmental stages	*GhMYB2*	psRNATarget prediction/ 5′-RACE	Ovule/fiber/leaf	↑	*Gossypium hirsutum*	asiRNA	Positive	[24]
Fiber-elongation; fiber-length	miR396b	Fiber elongation stage	*GhGRF5*	psRNATarget prediction/ degradome sequencing	Ovule/fiber/root/stem/leaf	↑	*Gossypium hirsutum*	STTM	Negative	[25]
Fiber-elongation	miR160a_A05	Fiber initiation-to-elongation stages	*GhARF17*	psRNATarget prediction/ 5′-RACE/ degradome sequencing	Ovule/fiber	↑	*Gossypium hirsutum*	STTM	Positive	[26]
Regulates the transition from fiber-cell elongation to secondary-cell-wall biosynthesis; fiber-elongation duration	miR319	Fiber initiation, elongation to secondary-cell-wall thickening stages	*GhTCP4*	psRNATarget prediction/ 5′-RACE/ degradome sequencing	Ovule/fiber	↑	*Gossypium hirsutum*	OE/RNAi	Positive	[27]

Note: "↑" indicates up-regulated expression, and "↓" indicates down-regulated expression.lated expression.

**Table 2:** miRNAs involved in stress response of cotton.

Stress Treatment	miRNA	Tissue	Target(s)	Assays for miRNA-Target Validation	Direction of miRNA Expression	Pathway	Species	Validation Method	Resistance Characteristics	References
*V. dahliae* stress	miR166b	Root/stem/leaf	*GhHB14*	psRNATarget prediction/ 5′-RACE/degradome sequencing	↑	SA signaling pathway	*Gossypium hirsutum*	VIGS	Positive	[36]
	miR171a	Root/stem/leaf	*GhSCL6*	psRNATarget prediction/ 5′-RACE	↓	SA signaling pathway	*Gossypium hirsutum*	VIGS	Negative	[37]
	miR319b	Root/stem/leaf	*GhTCP4* *-* *Like*	psRNATarget prediction/ 5′-RACE/degradome sequencing	↓	SA signaling pathway	*Gossypium hirsutum*	VIGS	Negative	[38]
	miR477	Root	*GhCBP60A*	psRNATarget prediction/ 5′-RACE/degradome sequencing	↑	SA signaling pathway	*Gossypium hirsutum*	VIGS	Positive	[39]
	miR482b	Root/stem/leaf/cotyledon	*GhRSG2*	psRNATarget prediction/degradome sequencing	↓	SA signaling pathway	*Gossypium hirsutum*	VIGS	Negative	[40]
	miR530	Leaf	*GhSAP6*	psRNATarget prediction/degradome sequencing	↓	SA signaling pathway	*Gossypium hirsutum*	VIGS	Negative	[41]
	miR7814	Root/stem/leaf/cotyledon	*GhCNL2*	psRNATarget prediction/ 5′-RACE	↓	SA signaling pathway/ROS	*Gossypium hirsutum*	VIGS	Negative	[42]
	miR164	Root	GhNAC100	psRNATarget prediction/ 5′-RACE/degradome sequencing	↑	JA signaling pathway	*Gossypium hirsutum*	VIGS	Positive	[43]
	miR398b	Root/hypocotyl/stem	*GhCSD1* */* *GhCSD2* */* *GhCCS1*	psRNATarget prediction/ 5′-RACE/degradome sequencing	↓	ROS homeostasis	*Gossypium hirsutum*	OE/VIGS	Negative	[44]
	miR482c	Root/stem/leaf	*GhRDUF4D*	psRNATarget prediction	↓	ROS burst/callose deposition	*Gossypium hirsutum/Nicotiana benthamiana/Arabidopsis thaliana*	VIGS	Negative	[45]
	miR157d	Leaf/rosette/inflorescence	*SPL13A/B*	psRNATarget prediction	↑	*/*	*/*	asMiRNA	Negative	[46]
	miR319c	Root/stem/leaf	*GhMYB33*	psRNATarget prediction/ 5′-RACE/degradome sequencing	↑	Anthocyanin accumulation	*Gossypium hirsutum/Nicotiana benthamiana*	CRISPR-Cas9/OE/VIGS	Positive	[47]
	miR397	Root/stem/leaf	*GhLAC4*	psRNATarget prediction/degradome sequencing	↓	Lignin biosynthesis pathway	*Gossypium hirsutum/Nicotiana benthamiana*	VIGS	Negative	[48]
*Fov* stress	miR5272a	Root/leaf/cotyledon	*GhMKK6*	psRNATarget prediction	↑	MAPK cascade signaling pathway	*Gossypium hirsutum/Nicotiana benthamiana*	VIGS	Negative	[49]
CLCuMuV stress	miR398	Leaf	/	Prediction	/	Virus defense pathway	*Gossypium hirsutum*	OE	Positive	[50]
	miR2950	Leaf	/	Prediction	/	Virus defense pathway	*Gossypium hirsutum*	OE	Positive	[50]
CLCrV stress	miR156b	Leaf	*Gh* *SPL*	Prediction	/	Virus defense pathway	*Gossypium hirsutum*	VIGS	Positive	[51]
Pest stress	miR390	Leaf	*GhTAS3*	psRNATarget prediction/degradome sequencing	↑	IAA/JA signaling pathway	*Gossypium hirsutum*	VIGS	Positive	[52]
Drought stress	miR477b	Root	*GhNAC1/GhSCL3*	psRNATarget prediction	↑	IAA signaling pathway	*Gossypium hirsutum*	VIGS	Negative	[53]
Salt stress	miR414c	Cotyledon/hypocotyl, root/stem/leaf	*GhFSD1*	psRNATarget prediction/ 5′-RACE	↓	ROS metabolism pathway	*Gossypium hirsutum/Nicotiana benthamiana/Arabidopsis thaliana*	VIGS/OE	Negative	[54]
	miRNVL5	Root/shoot/ovule/fiber	*GhCHR*	psRNATarget prediction/ 5′-RACE	↓	SOS1 pathway /K^+^/Na^+^ ratio	*Gossypium hirsutum/Arabidopsis thaliana*	OE	Negative	[55]
	miR390	Root/stem/leaf	*GhCEPR2*	psRNATarget prediction/ 5′-RACE/degradome sequencing	leaves: ↓ to ↑; stems: ↑; roots: ↑ to ↓	ABA signaling pathway	*Gossypium hirsutum/Arabidopsis thaliana*	VIGS/OE	Negative	[56]
HT stress	miR160	Anther	*GhARF10/17*	psRNATarget prediction/ 5′-RACE/degradome sequencing	↓	IAA signaling pathway	*Gossypium hirsutum*	OE	Negative	[57]
	miR156	Anther	*GhSPLs*	psRNATarget prediction/ 5′-RACE/degradome sequencing	↓/↑	IAA signaling pathway	*Gossypium hirsutum*	OE	Positive	[57]
	miR156	Mature pollen	*PERK12*	Prediction/degradome sequencing	↓	Receptor-like kinase-mediated heat-stress sensing pathway	*Gossypium hirsutum*	/	Positive	[58]
	miR8643	Mature pollen	*RBK1*	Prediction/degradome sequencing	↓	Receptor-like kinase-mediated heat-stress sensing pathway	*Gossypium hirsutum*	/	Positive	[58]
	miR167	Mature pollen	*Bp10*	Prediction/degradome sequencing	↑	ROS scavenging pathway	*Gossypium hirsutum*	/	Negative	[58]
	miR390	Mature pollen	*Bp10*	Prediction/degradome sequencing	↑	ROS scavenging pathway	*Gossypium hirsutum*	/	Negative	[58]
	miR167	Mature pollen	*ARF8*	Prediction/degradome sequencing	↑	Auxin signaling pathway	*Gossypium hirsutum*	/	Negative	[58]
Nutrient imbalance	miR156	Root	*SPLs*	Prediction	↑	Auxin signaling pathway	*Gossypium hirsutum*	/	Positive	[59]
	miR167	Root	*ARFs/IAR*_3_	Prediction	↑	Auxin signaling pathway	*Gossypium hirsutum*	/	Positive	[59]
	miR390	Root	*ARF_S_*	Prediction	↑	Auxin signaling pathway	*Gossypium hirsutum*	/	Positive	[59]
	miR393	Root	*TIR1/AFBs*	Prediction	↑	Auxin signaling pathway	*Gossypium hirsutum*	/	Positive	[59]
	miR171	Root	*SCL*	Prediction	↓	Auxin signaling pathway	*Gossypium hirsutum*	/	Negative	[59]
	miR396	Root	*GRFs*	Prediction	↓	Auxin signaling pathway	*Gossypium hirsutum*	/	Negative	[59]
	miRN44	Ovule	*GaZIP1L*	psRNATarget prediction/ 5′-RACE	↓	Zn^2+^ accumulation	*Gossypium arboreum/Arabidopsis thaliana*	OE	Positive	[60]
	miR408	Leaf	*/*	Stable overexpression phenotype	*/*	Copper-transport/antioxidant response pathway	*Gossypium hirsutum/Arabidopsis thaliana*	OE	Positive	[61]

Note: "↑" indicates up-regulated expression, and "↓" indicates down-regulated expression.

## Data Availability

No new data were created or analyzed in this study. Data sharing is not applicable to this article.
